# Quality of life among female childhood cancer survivors with and without premature ovarian insufficiency

**DOI:** 10.1007/s11764-021-00987-y

**Published:** 2021-01-19

**Authors:** Hjelmér Ida, Gustafsson Kylberg Alicia, Fridenborg Anna, Leijonhufvud Irene, Nyström Anna, Mörse Helena, Elfving Maria, Henic Emir, Nenonen Hannah

**Affiliations:** 1 Department of Translational Medicine, Molecular Genetic Reproductive Medicine, Clinical Research Centre, Lund University, Malmo, Sweden; 2 Department of Translational Medicine, Reproductive Medicine Centre, Skåne University Hospital, Lund University, Malmo, Sweden; 3 Department of Pediatrics, Pediatric Endocrinology, Skåne University Hospital, Lund University, Lund, Sweden; 4 Department of Pediatrics, Pediatric Oncology and Hematology, Skåne University Hospital, Lund University, Lund, Sweden

**Keywords:** Childhood cancer survivors, Female, Premature ovarian insufficiency, Quality of life

## Abstract

**Purpose:**

Due to an increase in survival, a growing population of childhood cancer survivors (CCS) is present. However, female CCS are at risk of developing premature ovarian insufficiency (POI) after cancer treatment. POI involves a decreased chance of conceiving and the increased infertility state has a large impact on affected individuals’ health and mental life. The objective of this study was to investigate health state and well-being among female CCS with and without POI and healthy controls (HC).

**Methods:**

Female CCS treated in southern Sweden between 1964 and 2008 were included. Each patient was matched with a HC. The final study population included 167 female CCS and 164 HC that were examined between October 2010 and January 2015 at the Reproductive Medicine Centre at Skåne University Hospital in Malmö, Sweden. All participants, except for two HCs, answered an EQ-5D-3L questionnaire for measuring health state including a visual analogue scale (VAS) for estimating well-being.

**Results:**

There were 22 CCS with POI, none of the HC had POI. The mean health state differed among groups (unadjusted: *P* = 0.002; adjusted: *P* = 0.007). A difference in mean experienced well-being among groups was noted (unadjusted: *P* = 0.003; adjusted: *P* = 0.012). Lowest well-being was found in the CCS group with POI (*P* = 0.024).

**Conclusions:**

Female CCS have a significantly decreased health state and well-being. Female CCS with POI additionally have the lowest self-estimated well-being.

**Implications for Cancer Survivors:**

Female CCS with POI should be identified early in order to give them adequate information and support.

## Introduction

Today, the survival rate in Sweden for childhood cancer is 85% [1], resulting in an adult population of individuals that have survived cancer. The treatment regimens include either surgery, chemotherapy or radiation or a combination thereof. It is estimated that two-thirds of CCS will experience at least one treatment-related health condition [2–4]. One complication that can occur is premature ovarian insufficiency (POI), because radiation with ovaries in the field or chemotherapy, especially with alkylating agents, may have detrimental effects on the ovarian follicles [5–7]. The prevalence of POI among female childhood cancer survivors (CCS) is approximately 8–13% [7, 8], as compared with ~ 1% in the general population [9].

The definition of POI is follicular depletion or ovarian dysfunction before the age of 40, but the symptoms may be highly individual due to the severity of the gonadal dysfunction and the age of the affected individual [10]. In general, affected individuals may present with amenorrhea or delayed puberty, but POI can also manifest as a prolonged time-to-pregnancy or as the use of assisted reproduction technologies (ART). Diagnosis is based on elevated serum follicle-stimulating hormone (FSH) levels and decreased serum oestradiol levels [11].

Women diagnosed with POI are offered a hormone replacement therapy with administration of exogenous oestrogen to prevent the harmful physiological effects of low endogenous serum oestradiol. For those that wish for a child, the prognosis of conceiving spontaneously is 5–10% for POI cases, and therefore, the use of an egg donor is often recommended [12].

The infertility state that POI leads limits the ability to have children and has a large impact on the affected individuals’ health and mental life [13]. Population-based studies have shown that cancer survivors generally are less likely to have biological children compared with controls without a history of cancer [14, 15], and also that they have an increased risk of delivery complications [16]. It has previously been shown that cancer survivors worry about the risk of being infertile, and the risk of infertility among survivors also affected partner relationships [17]. Although cancer survivors had a strong desire for having children [15, 18], they were worrying about the health of a future biological child [19, 20] as well as their own health [21]. Additionally, several studies have confirmed a link between POI and quality of life (QoL) and depression [22, 23]. The objective of this study was therefore to investigate QoL and health state (as defined by the EQ-5D-3L questionnaire) [24–26] among female CCS with and without POI and healthy controls (HC). When planning this study, we also hypothesized that women who wished to have children but could not have them would score worse overall in the EQ-5D-3L than women who had been able to get as many children as desired.

The EQ-5D-3L questionnaire is a self-assessment descriptive system with focus on the participants’ health-related quality of life [27]. The EQ-5D-3L questionnaire is composed of two parts. The first part contains five questions considering mobility, hygiene/self-care, basic activities, pain, and fear/anxiety. Each question can be answered by ticking one of three boxes where 1 equals no problem, 2 some problems, and 3 equals extreme problems, giving 243 possible health states. The answers to these questions can be converted to a single index number [27], reflecting the health state of the individual according to the preferences of the general population (valuation cohort) of the country or region the participant belongs to. The index numbers in the valuation cohort, termed value sets, can be extracted with either the time to trade off (TTO) valuation technique or the VAS valuation technique [27, 28]. The questionnaire also includes a visual analogue scale (VAS) for recording the subjects’ self-rated health on the current day on a scale ranging from 0 to 100 where the top endpoint is ‘Best imaginable health state’ and bottom is ‘Worst imaginable health state’. The ratings can be used as a quantitative measure of health within the examined group. The EQ 5D 3L method is well validated and there are at least 35 different national validity populations that can be used as reference when analysing results. In the Swedish value set, the answer “no problem” to each of the five questions gives an index score for TTO of 0.9694 and VAS of 88.86 whereas the opposite answer “extreme problems” gives a TTO of 0.3403 and VAS of 17.24 [29].

The EQ-5D-3L questionnaire is used within academia and the pharmaceutical industry around the world. In clinical trials, it is used, e.g. to evaluate the efficacy and safety of drugs [28] in relation to their impact on health-related quality of life for the participants during the trial. As an example to show the broad use of the EQ-5D from Sweden, where our study took place, there are 105 Swedish National Quality Registries, and in 46 of them, there is patient-reported outcome measures reported with the EQ-5D method [30]. The registries where data is collected include, e.g. the musculoskeletal system, cancer, psychiatry and endocrine organs. A majority of these registries use the results to give feedback to patients or clinicians on a group level but some also provide it to treating clinicians or individual patients. Mostly, the data is used for assessing interventions, quality indicators and for following up the quality of care [30]. The EQ-5D-3L has also been used in previous studies on cancer survivors and late toxicity effects after cancer treatment [31–34]. In the study by Yu et al. on breast cancer survivors in Korea, there was no difference in the EQ-5D-3L index in general between survivors and an age-matched control group 5 years after surgery; however, in the categories of pain/discomfort and anxiety/depression, the survivors still scored worse than the controls [34]. In a study on long-term survivors of nasopharyngeal cancer including both men and women, quality of life was measured with the EQ-5D-3L and related to type of treatment [31]; it was found that age and type of treatment affected the overall quality of life.

If lowered QoL and decreased health state could be recognized among female CCS with infertility difficulties, care could be taken at an early stage by health care professionals in order to give these women adequate support and counselling.

## Subjects and methods

### Participants

The study population has previously been described by Nyström et al. [8]. Summarily, the inclusion criteria were females treated for childhood cancer in southern Sweden between the years 1964 and 2008. The participants were below 18 years of age at cancer diagnosis and had completed treatment more than 2 years before inclusion to the study. The exclusion criteria were females with rare tumours or with focal tumours outside of the central nervous system treated with surgery only. Each patient was matched with a HC from the southern region in Sweden regarding sex, date of birth, ethnicity and smoking habits through the Swedish Population Registry. The study was approved by the regional ethical committee board in Lund, Sweden (approval no. 523/2009). A written informed consent was obtained from all participants before study inclusion.

The final study population included 167 female CCS and 164 female HC that were examined between October 2010 and January 2015 at the Reproductive Medicine Centre (RMC) at Skåne University Hospital in Malmö, Sweden.

Premature ovarian insufficiency was diagnosed in women with primary or secondary amenorrhea before 40 years of age, prior to hormone replacement therapy (HRT) and very low or undetectable anti-Müllerian hormone (AMH). Because almost every woman with ovarian insufficiency was on hormone replacement therapy at the time of examination, oestradiol and FSH values could not be used for defining POI.

Five CCS with POI due to hypothalamic/pituitary dysfunction, including patients treated for craniopharyngioma, were excluded from all analyses, since severe hypothalamic damage often has a high impact on QoL.

## Methods

All participants, except for two HCs, answered an EQ-5D-3L [24–26] questionnaire including a visual analogue scale (VAS) (registration number: 35268; EuroQol Research Foundation, Rotterdam, The Netherlands) and one questionnaire regarding medical history. Participants with missing data were excluded. Data collected from the questionnaires on medical history included for example presence of primary or secondary amenorrhea, use of contraceptives or oestrogen substitutes, marital status/domestic partnership, wish for a child, number of pregnancies and adoptions (Table 1).

**Table 1:** Clinical parameters among CCS without POI, CCS with POI and HC

	CCS w/o POI *n* = 140	CCS w POI *n* = 22	HC *n* = 162	*P*^a^
Age at examination (yr)	33.6 (19–58)	38.9 (22–56)	35.0 (19–58)	0.031
BMI (kg/m^2^)	25.1 (16.2–43.8)	23.4 (17.7–34.1)	19.8 (14.5–29.6)	< 0.000
FSH (IU/L)	10.5 (0.0–124.0)	41.4 (0.6–131.0)	9.1 (0.2–109.0)	< 0.000
Oestradiol (pmol/L)	178.8 (17–1342)	206.0 (25–1020)	162.9 (15–1232)	0.511
HRT	0	*n* = 15 (68%)	0	< 0.000
Biological children (mean)	1.05 (0–5)	0.19 (0–2)	1.10 (0–5)	0.005
Adopted children (mean)	0 (0–0)	0.18 (0–1)	0.01 (0–1)	< 0.000
ART	*n* = 9 (6%)	*n* = 8 (36%)	*n* = 12 (7%)	< 0.000
Married/domestic partnership	*n* = 80 (57%)	*n* = 13 (59%)	*n* = 104 (64%)	0.450

*ART* assisted reproductive technology, *CCS* childhood cancer survivors, *HC* healthy controls, *HRT* hormone replacement therapy, *IU* international units, *w* with, *w/o* without, *yr* year

^a^Comparison among the three groups (CCS w/o POI, CCS w POI, HC) with the univariate analysis of variance or Chi^2^ test

Health states for each participant were extracted from the EQ-5D-3L and converted into a single summary index called time trade off (TTO), extracted from a Swedish population value set [29].

### Hormonal analyses

For endocrine serum analysis, fasting blood samples were drawn early in follicular phase of the menstrual cycle in those women with a regular menstrual cycle and on any day for those not having a regular menstrual cycle or for those on HRT, as described by Nyström et al. [8]. Plasma levels of FSH (Roche 11775863, Mannheim, Germany) were measured at the Department of Clinical Chemistry, Skåne University Hospital in Lund, Sweden, with a detection limit of 0.2 IU/L. All FSH values < 0.2 IU/L were stated as 0. An update of methods regarding the FSH analysis occurred during the course of the study, and FSH values obtained with the old method were transformed for the most recent assay as described by Bobjer et al. [35]. Plasma levels of oestradiol (DELFIA PerkinElmer Inc., Wallac Oy, Turku, Finland) were measured at the Department of Clinical Chemistry, Skåne University Hospital in Malmö, Sweden, with a detection limit of < 10 pmol/L. The method was improved with a detection limit of 3 pmol/L (LC-MSMS, NPU09357) during the course of the study. All E2 values < 10 pmol/L were stated as 0.

### Statistical analysis

Comparisons of age at examination, BMI, baseline hormones and number of children among groups (CCS without POI, CCS with POI and HC) were carried out using a univariate analysis of variance. Five CCS with POI were excluded due to hypothalamic/pituitary dysfunction from all comparisons between groups. Comparisons of incidence of HRT, incidence of ART and marital status/domestic partnership among groups were carried out using a Chi^2^ test.

The POI group was analysed as one group including all 22 subjects when comparing them with CCS without POI and HC. Nevertheless, it consisted of two subgroups, women that were diagnosed with primary amenorrhea or amenorrhea before 20 years of age and those who were diagnosed with amenorrhea at ages ranging from 20 to 39 years. The average summary index from the EQ-5D-3L (TTO) value and EQ-VAS was compared between these groups with the two sample *t* test assuming unequal variances. Median values of the summary index from the EQ-5D-3L and the EQ-VAS for CCS without POI, CCS with POI and HC were compared using the univariate analysis of variance. In the same way, the total group of CCS was compared with the HC.

The values for TTO and VAS-score were evaluated within each group for equal variances using the Shapiro-Wilk test. The relation between TTO value, VAS score and groups was analysed with the univariate analysis of variance with a relevant post hoc test and a linear regression in an unadjusted analysis and with adjustment for the following covariates: age at examination, children (yes/no), wish for a child at examination, wish for a child in the future and marital status/domestic partnership. These factors were chosen for adjustment because they could have an influence on the answers given to the questions that create the TTO and VAS score. The same method was used to compare the answers between women who had the number of children they wished for and those who did not, but in this case, the covariates were only age at examination and marital status/domestic partnership.

Statistical analyses were performed with IBM Statistical Package for the Social Sciences (SPSS, Inc., Chicago, IL, USA) version 25. A *P* value < 0.05 was considered statistically significant and confidence intervals were set to 95%.

## Results

### Clinical parameters

When analysing clinical parameters, 140 CCS without POI were available, as were 22 CCS with POI and 162 HC.

There was a difference in age at examination between the three groups (CCS without POI, CCS with POI and HC; *P* = 0.031; Table 1) and in BMI (*P* < 0.000; Table 1). However, when analysing the total CCS group compared with the HC, the age difference was no longer evident (CCS: 34.3 years, HC: 35.0 years; *P* = 0.454). A difference in FSH among groups (CCS without POI, CCS with POI and HC) was also noted (*P* < 0.000), which also was true for HRT (*P* < 0.000; Phi and Cramer’s V: 0.816). The POI group also had fewer biological children compared with the other groups (*P* = 0.005). Adoption was more common within the POI group (*P* < 0.000), as was use of ART (*P* < 0.000; Phi and Cramer’s V: 0.260). There were no differences in basal oestradiol levels or marital status/domestic partnership among groups (Table 1). Treatment in the patient cohort is demonstrated in Table 2. The POI group was treated more frequently with irradiation and chemotherapy, but for cranial irradiation, the numbers were similar. The average time since amenorrhea (POI diagnosis) for the POI group was 16 years, ranging from 1 to 23 years in women with secondary amenorrhea and 15–34 years in women with primary amenorrhea or amenorrhea before 20 years of age.

**Table 2:** Treatment in the cohort of childhood cancer survivors (CCS) and CCS with premature ovarian insufficiency (POI). Patients with hypothalamic/pituitary cause of POI excluded

Type of treatment	CCS *n* = 140 (%)	CCS with POI *n* = 22 (%)
All radiotherapy	65 (46)	17 (77)
Cranial irradiation	41 (29)	7 (23)
Abdominal irradiation	17 (12)	16 (73)
Both cranial and abdominal irradiation	9 (6)	7 (32)
TBI	2 (1)	5 (23)
All chemotherapy	104 (74)	20 (91)
Alkylating agent	65 (46)	14 (64)
HSCT	4 (3)	7 (23)
Only surgery	19 (14)	0

Notes: Data presented as number and percent

*HSCT* hematopoietic stem cell transplantation, *TBI* total body irradiation

### EQ-5D-3L answers

Due to missing data, 138 CCS without POI were available when analysing EQ-5D-3L answers, as were 22 CCS with POI and 159 HC.

**Fig. 1:**
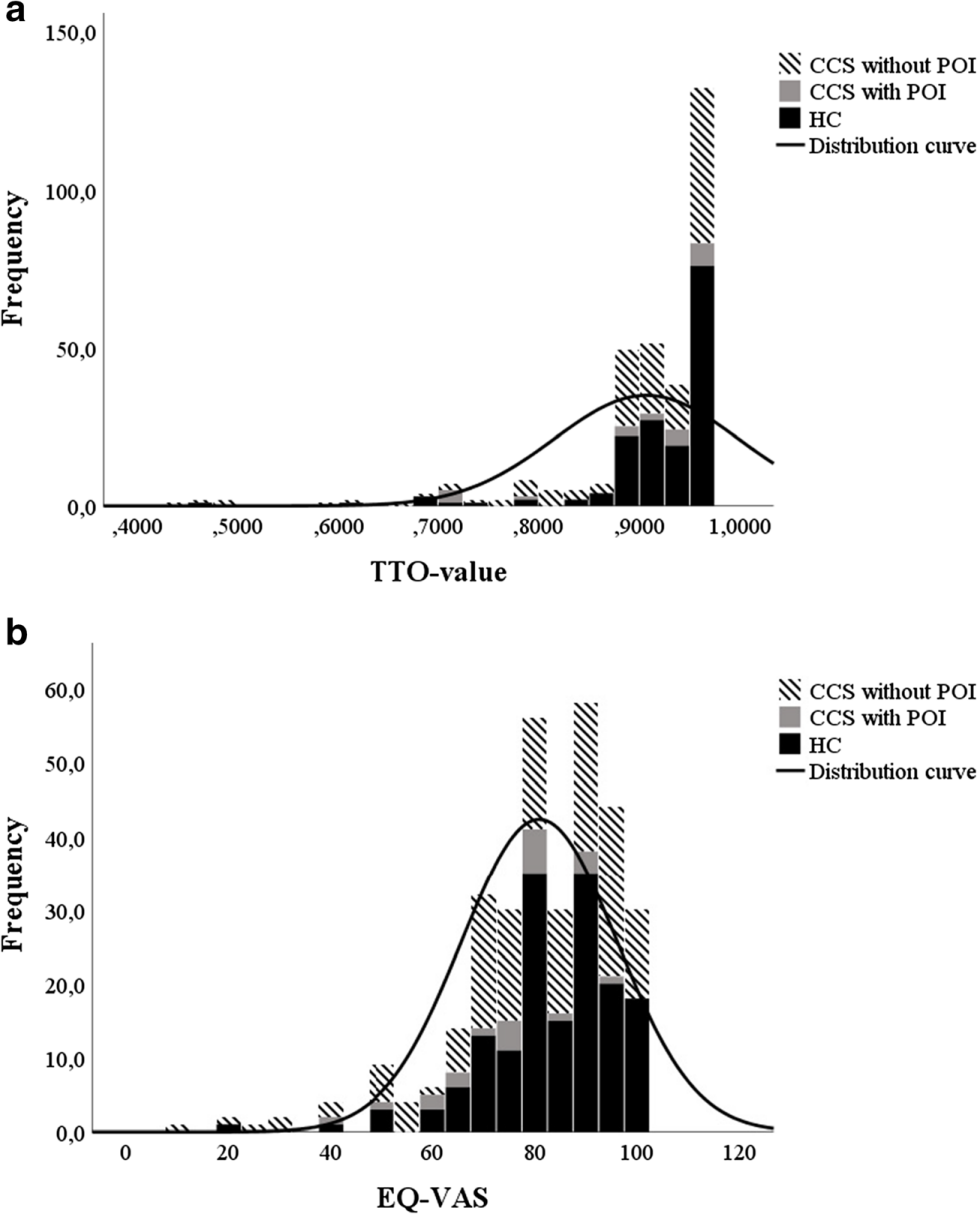
**a** Mean health state, presented as TTO values, among the CCS group without POI, CCS group with POI and the HC. Line represents distribution curve. Mean TTO value for CCS without POI: 0.8913 ± 0.1064; mean TTO value for CCS with POI: 0.8892 ± 0.0956; mean TTO value for HC: 0.9230 ± 0.0728. CCS: childhood cancer survivor. POI: premature ovarian insufficiency. HC: healthy controls. TTO: time trade-off. **b** Well-being, defined as EQ-VAS answers, among CCS without POI, CCS with POI and HC. Line represents distribution curve. Mean EQ-VAS for CCS without POI: 79.0 ± 17.6; mean EQ-VAS for CCS with POI: 74.5 ± 13.3; mean EQ-VAS for HC: 83.4 ± 12.6. CCS: childhood cancer survivor. POI: premature ovarian insufficiency. HC: healthy controls. VAS: visual analogue scale

The mean health state, presented as TTO values, differed among the CCS group without POI, CCS with POI and the HC group (unadjusted: *P* = 0.010; adjusted: *P* = 0.027; Table 3; Fig. 1a). However, in the unadjusted analysis, the difference in mean TTO values was between the CCS group without POI and the HC group, after post hoc correction. When CCS without POI and CCS with POI was processed as one group and compared with HC, there was a significant difference (0.8910 ± 0.1047 vs. 0.9230 ± 0.0728; unadjusted: *P* = 0.002; adjusted: *P* = 0.007), demonstrating a lower health state for CCS compared with HC.

**Table 3:** Mean health state, presented as TTO values, and well-being, defined as EQ-VAS answers, among CCS without POI, CCS with POI and HC

	CCS w/o POI *n* = 138^a^/140^b^	CCS w POI *n* = 22	HC *n* = 159^a^/161^b^	*P*^c, d^	*P*^c, e^
TTO value	0.8913 ± 0.1064	0.8892 ± 0.0956	0.9230 ± 0.0728	0.010	0.027
EQ-VAS	79.0 ± 17.6	74.5 ± 13.3	83.4 ± 12.6	0.037^f^, 0.024^g^	0.017

*CCS* childhood cancer survivors, *HC* healthy controls, *TTO* time trade-off, *VAS* visual analogue scale, *w* with, *w/o* without

^a^*n* in TTO analysis

^b^*n* in EQ-VAS analysis

^c^Comparison among groups (CCS w/o POI, CCS w POI, HC)

^d^Analysis including post hoc

^e^Adjusted *P* values

^f^CCS w/o POI compared with HC

^g^CCS w POI compared with HC

A linear significant association with TTO value and CCS without POI, CCS with POI and HC was also noted (unadjusted: *B* = 0.016, 95% CI: [0.006, 0.026], *P* = 0.003; adjusted: *B* = 0.014, 95% CI: [0.004, 0.024], *P* = 0.008).

Factors adjusted for were age at examination, children (yes/no), wish for a child at examination, wish for a child in the future and marital status/domestic partnership.

## EQ-VAS answers

Due to missing data, 140 CCS without POI were available when analysing EQ-VAS answers, as were 22 CCS with POI and 161 HC.

Differences in mean experienced well-being, defined as EQ-VAS answers, among CCS without POI and CCS with POI compared with HC were noted (unadjusted: *P* = 0.037 for CCS without POI and *P* = 0.024 for CCS with POI; Table 3; Fig. 1b). In the adjusted analysis, a difference among the three groups was noted (adjusted: *P* = 0.017; Table 3; Fig. 1b). When all CCS was compared with the HC group, the EQ-VAS was lower in the CCS group compared with HC (78.4 ± 17.1 vs. 83.4 ± 12.6; unadjusted: *P* = 0.003; adjusted: *P* = 0.012), demonstrating a reduced experienced well-being among CCS compared with HC.

A linear significant association with EQ-VAS score and CCS without POI, CCS with POI and HC was also noted (unadjusted: *B* = 2.25, 95% CI: [0.533, 3.96], *P* = 0.010; adjusted: *B* = 1.81, 95% CI: [0.130, 3.49], *P* = 0.035). Factors adjusted for were age at examination, children (yes/no), wish for a child at examination, wish for a child in the future and marital status/domestic partnership.

### Analysis within the POI group

The group of CCS with POI contained two subgroups, 13 women that were diagnosed with primary amenorrhea or amenorrhea before 20 years of age and 9 women who were diagnosed with amenorrhea at ages ranging from 20 to 39 years (secondary amenorrhea). The average TTO value and EQ-VAS did not differ between these two subgroups (TTO; primary amenorrhea 0.883 ± 0.1 vs. secondary amenorrhea 0.898 ± 0.1; *P* = 0.730) (EQ-VAS; primary amenorrhea 74 ± 14.0 vs. secondary amenorrhea 75 ± 13.87; *P* = 0.873).

### Current wish for a child and the influence on TTO and EQ-VAS

The two groups used for this analysis were women who had the number of children they wished for and the other group with those who had given one of the following answers to the question of current wish for children: planning to have biological children in the future, not able to have children of my own (no ovarian function), not able to have children (uterus not able to carry a pregnancy), partner not able to have children, partner does not want children, trying to become pregnant since < 2 years, trying to conceive > 2 years, I think I am too old/I am too old (otherwise I would like to have a child).

A total of 118 women had the number of children they wished for (group CWY) and 167 gave one of the alternative answers stated above (group CWN). The question of “wishing to have children” had never been relevant for 39 participants; they were excluded from this analysis. No statistically significant difference in mean health state (TTO) or in experienced well-being (EQ-VAS) was found between the groups (TTO; CWY 0.91 ± 0.10 vs. CWN 0.91 ± 0.08; unadjusted: *P* = 0.524; adjusted: *P* = 0.551) (EQ-VAS; CWY 81.14 ± 16.5 vs CWN 80.7 ± 14.8; unadjusted: *P* = 0.705; adjusted: *P* = 0.227). Factors adjusted for were age at examination and marital status/domestic partnership.

## Discussion

This study aimed to highlight the health state and well-being of female CCS diagnosed with POI, and we found that these women have a significantly decreased self-estimated well-being compared with CCS without POI and healthy controls regarding EQ-VAS. When analysing only the POI group, there was no difference in health state (TTO) compared with healthy controls; this could be due to the small number in the POI group. However, when analysing the CCS group in total, a difference was evident. The actual difference in TTO values between the groups is small and may not reflect an overall clinical significant difference but these analyses are group based and the individual differences should not be ignored when working with these patient groups. Our result is in line with previous studies on the same subject [13, 22, 23]. However, contradictory results have also been published, where long-time cancer survivors in the USA scored higher on well-being measures as self-reported health, utility and happiness [36]. National and methodological discrepancies may account for this difference, but it is worth mentioning that long-term cancer survivors may score higher than a control population on well-being. This may be something to be aware of when counselling these patients and indicates that long-term cancer survivors may appreciate their health status more than the general population. The POI patients’ expectations and later experience of well-being may also be dependent on the information regarding expected fertility given to them in connection with their treatments. This could be demonstrated by the finding that there was no difference in TTO or EQ-VAS depending on age at POI diagnosis. However, the compared groups were small and a larger cohort with POI could give a more robust result.

The results from the EQ-VAS were in line with results from a previous larger population study [37], where the mean EQ-VAS was 81.0 for Swedish women aged 21–34 years and 80.0 for those aged 35–44. In our present study, healthy controls with an average age of 35 had an EQ-VAS score of 83.4, and the CCS without POI with an average age of 33.6 scored 79 on the EQ-VAS. These results show that the HC and CCS women in this study score close to the general female population on the EQ-VAS. The women with POI on the other hand have a lower EQ-VAS 74.5 with an average age of 38.9 years. Even in comparison with women aged 45–54 years from the general population with an EQ-VAS of 79.0, the POI women score low. The results from the present study may correspond to the actual population in regard to the HC. One way to interpret these results would be that female CCS have a high quality of life in general but they still have a lower quality of life than women who have not had childhood cancer.

When relating the EQ-VAS scale to the index TTO values of the EQ-5D-3L questionnaire, there were discrepancies between the EQ-VAS score and the answers of the questionnaire. The index TTO value found in the questionnaire was different from the related EQ-VAS scale index. This has been found previously and indicates that the EQ-VAS scale measures something else than the questions. You could argue that the EQ-VAS scale is a method that indicates the well-being right this minute and the questionnaire can deal with events that have happened some time ago. Our study on CCS indicates that the discrepancies between the TTO value and the EQ-VAS scale often are on the positive side. Participants (CCS) with a TTO value that corresponds to a lower EQ-VAS score in the index study [29] more often indicate a higher EQ-VAS score. If this has to do with a more general positive feeling about life due to their past disease is still unknown. It could also indicate that they are more worse off than the health care realizes and should be given even more help, support and counselling than they have received so far.

When planning this study, we hypothesized that women who wanted children but could not have them would score worse in the EQ-5D-3L than women who had been able to get as many children as desired. This hypothesis was rejected; women who had the number of children they wished for did not score higher than women who had not. This result was surprising considering the amount of literature showing that childlessness can be a heavy burden for women to carry [38–41]. On the other hand, our study did not only include infertile women, and when the question was posed, there was still a possibility that women in the group still wanting children could have children in the future. The fact that there was no difference could also reflect the possibility that the participants had understood and accepted the difficulties related to their wish for having children.

We found that women with POI had fewer children, which was expected considering their impaired reproductional function (Table 1). They also used ART to a higher extent than other groups; this could be regarded as positive as it shows that they are provided with and use the resources that are available for managing their impaired fertility. Hopefully today and in the future, more women could be helped by these resources considering that they are more available and that the ART methods are refined continuously.

Childhood cancer survivors in general have an increased risk of psychological distress with female gender, lower education level and lower income as risk factors [42]. Different cancer treatments are also of importance; cranial irradiation with possible influence on cognitive function is associated with lower QoL in several studies [42–45]. In the POI group in our cohort, slightly fewer were treated with cranial irradiation. Abdominal irradiation, TBI, HSCT and chemotherapy were as expected more frequently, since these treatments are associated with a higher risk of ovarian dysfunction. Considering that the treatment given to the POI group is not exactly the same as to the CCS, we cannot exclude the impact of different treatments on QoL. Cranial irradiation is associated with lower QoL [46], and the disease and also chemotherapy-related late effects can also be of importance.

The strengths of this study are the size of the cohorts and the method using age-matched controls. Additionally, there is a lot of data collected in relation to the women’s reproductive function and their family wishes (Table 1) presented in more detail in Nyström et al. [8]; so, our hypothesis could be appropriately tested. One limitation in this study is that the definition of POI was slightly modified compared with the original definition where levels of FSH and oestradiol should be considered before diagnosis. In summary, in this study on Swedish female CCS, CCS had lower health state and well-being compared with controls. Those in the CCS group that developed POI had the lowest self-estimated well-being of all groups. However, on a group level, the total CCS group scored their self-esteemed quality of life relatively high compared with HC. This may indicate that support offered to them has improved their quality of life. Therefore, it is even more important that special care and counselling should be offered in order to give women with POI even more adequate support.
